# Supplementary material for: Late Pleistocene Altitudinal Segregation and Demography Define Future Climate Change Distribution of the Peromyscus mexicanus Species Group: Conservation Implications
Source: Animals (Basel). 2023 May 25;13(11):1753. doi: 10.3390/ani13111753 (PMC10251973; doi:10.3390/ani13111753)
Supplement: Supplementary file 1 [file animals-13-01753-s001.zip › animals-2339328-supplementary.pdf]

Late Pleistocene altitudinal segregation and demography define future climate change distribution of the *Peromyscus mexicanus* species group: conservation implications

Sergio G. Pérez-Consuegra, Laura Sánchez-Tovar, Gerardo Rodríguez-Tapia, Susette Castañeda-Rico, Ella Vázquez-Domínguez

Supplementary material

Table S1 - Table S3
Figure S1 - Figure S10

Table S1. List of the 18 samples of the *Peromyscus mexicanus* group that amplified for a fragment of the Growth Hormone Receptor (GHR; 829 bp); species (lineage), locality and ID of the museum collection it was obtained from are indicated for each sample. GenBank Accession numbers for the haplotypes obtained.

**ID**	**Species**	**lineage**	**locality**	**ID museum collection**	**GenBank Accession number**
2SGGU	*Peromyscus tropicalis*	B	San Gil, Guatemala	CM 117024	OQ992674
118MiVE	*Peromyscus mexicanus*	C	Misantla, Veracruz	MZFC 11177	OQ992671
119TuVE	*Peromyscus mexicanus*	C	Tutotepeq, Veracruz	MZFC 8995	OQ992686
81PoGU	*Peromyscus gymnotis*	D	Pochuta, Guatemala	USAC 5039	OQ992682
29GrCH	*Peromyscus cf zarhynchus*	F	Montebello, Guatemala	ECO 1216	OQ992677
27GrCH	*Peromyscus cf zarhynchus*	F	Montebello, Guatemala	ECO 1427	OQ992685
38YaGU	*Peromyscus cf zarhynchus*	G	Yalambojoch, Guatemala	USAC 4758	OQ992673
39IxGU	*Peromyscus cf zarhynchus*	G	Ixcansan, Guatemala	USAC 4750	OQ992678
55xtGU	*Peromyscus guatemalensis*	H	Ixtatan, Guatemala	MVZ 223209 (USAC 4896)	OQ992676
49LjGU	*Peromyscus guatemalensis*	H	LajChimel, Guatemala	USAC 5122	OQ992670
65SMGU	*Peromyscus cf guatemalensis*	I	San Marcos, Guatemala	USAC 5001	OQ992684
77GeGU	*Peromyscus cf guatemalensis*	J	Georginas, Guatemala	USAC 4643	OQ992679
78GeGU	*Peromyscus cf guatemalensis*	J	Georginas, Guatemala	USAC 4644	OQ992681
1CaGU	*Peromyscus grandis*	K	Carchá, Guatemala	MVZ 227028 (USAC 5151)	OQ992672
94MoES	*Peromyscus salvadorensis*	M	Morazan, El Salvador	USAC 5063	OQ992683
17AgHO	*Peromyscus nicaraguae*	N	Agalta, Honduras	CM 113172	OQ992675
70PiGU	*Peromyscus cf guatemalensis*	O	Pinalon, Guatemala	USAC 4409	OQ992680
71PiGU	*Peromyscus cf guatemalensis*	O	Pinalon, Guatemala	USAC 4411	OQ992687

Table S2. Six lineages selected from the *Peromyscus mexicanus* group for the potential future distribution modeling.

***Peromyscus mexicanus* (lineage C)**<br>Distribution: from *ca*. 500 to 1700 m in mountains along the Sierra Madre Oriental in Mexico, from Hidalgo to Veracruz and Oaxaca states, neighboring the canyon of the Valle de Tehuacán.<br><br>Vegetation type: tropical montane cloud forest, high annual rainfall, with steep mountain slopes below the freezing (fog) elevation band.<br>Linage is isolated from *P. gymnotis* by the lowlands of Veracruz and the Isthmus of Tehuantepec. In central Mexico, the montane cloud forest at higher elevations is occupied by *Peromyscus furvus*, with a potential sympatry zone.
***Peromyscus guatemalensis* (lineage H)**<br>Distribution: from 1700 up to 3200 m at cold plateaus of the Sierra de los Cuchumatanes in Guatemala and relatively humid highlands of the Sierra Madre de Chiapas (El Triunfo) and Guatemala. High annual precipitation (> 1500 mm), cold temperatures above freezing elevation band, and with steep slopes. Lineage geographic limits in central Guatemala are not clear and can be mistaken with *P. phylombrius*.<br><br>Vegetation type: broad-leaf forest or cold and high elevation montane cloud forest on the Sierra Madre de Chiapas (El Triunfo) and Guatemala (San Marcos y neighboring Quetzaltenango); the eastern limits are not clearly defined.
***Peromyscus salvadorensis* (lineage M)**<br>Distribution: mountain lowlands near sea level to approximately 2500 m, along a region dominated by dry valleys; the lineage distribution is restricted to rainy lowland zones (Suchitán, Trifinio, Celaque volcanoes, and Sierra de Santa Bárbara (west of Central Honduras Depression) to Cacahuatique mountain in El Salvador.<br><br>Vegetation type: broad-leaf forest and evergreen forest; some sones with semi-deciduous and pine forest; summer rains and strong winds; steep slopes.
***Peromyscus zarhynchus* (lineage E)**<br>Distribution: from 1000 to 2900 m along the Altos de Chiapas; its limit on the Sierra de Cuchumatanes in Guatemala (Huehuetenango and Quiché) is at 1700 m, likely by competitive exclusion with *P. guatemalensis*, which is parapatric on these mountains.<br><br>Vegetation type: broad-leaf and evergreen forests, with extreme annual precipitation regimes (can reach 7000 mm); steep slopes.
***Peromyscus gymnotis* (lineage D)**<br>Distribution: mountain lowlands near sea level to 1675 m along the southern Pacific coast of

Chiapas and Guatemala, the region of Chimalapas in Oaxaca and the low mountains of northern Altos de Chiapas (e.g., Palenque).<br><br>Vegetation type: broad-leaf and evergreen forests, with extreme precipitation regimes. Low elevation mountains are more similar to mountain tropical rain forests, while the highlands are dominated by low elevation cloud forest, with > 1500 mm annual rainfall, steep slopes below the frost line.
Peromyscus nicaraguae **(lineage N)**<br>Distribution: mountain lowlands near sea level to approximately 2000 m. Mountains to the East of the Depresión Central de Honduras along the Atlantic versant, and to the North of Honduras along a little higher and more rainy mountains. It is also found near Matagalpa, Nicaragua on > 1000 m mountains, and likely reaching small mountain chains on the Costa Rica border.<br><br>Vegetation type: broad-leaf and evergreen forests, with extreme precipitation regimes. Low elevation mountains are more similar to mountain tropical rain forests, while the highlands are dominated by low elevation cloud forest, with steep slopes.

Table S3. Current and future potential distribution ranges (in km^2) based on scenario A2 (SREASA2) and scenario B1 (SRESB1), which consider medium-high and low-medium emissions, respectively. Future projections were done considering the general circulation model cccma_cgcm3_1_t47 for three time periods (2020, 2050, 2080).

**Lineage**		**Scenario A2**			**Scenario B1**		
	**current**	**2020**	**2050**	**2080**	**2020**	**2050**	**2080**
P. mexicanus	8,246	13,866	12,185	13,985	5,948	11,202	10,903
P. gymnotis	51,063	23,335	26,561	34,312	28,671	15,800	52,669
P. nicaraguae	59,521	11,305	19,066	24,356	29,934	27,599	20,314
P. guatemalensis	12,918	9,159	10,056	9,479	10,320	7,698	12,656
P. salvadorensis	17,613	32,037	41,898	19,318	26,577	28,608	26,790
P. zarhynchus	3,095	2,473	2,910	3,870	3,269	2,285	3,715

a)

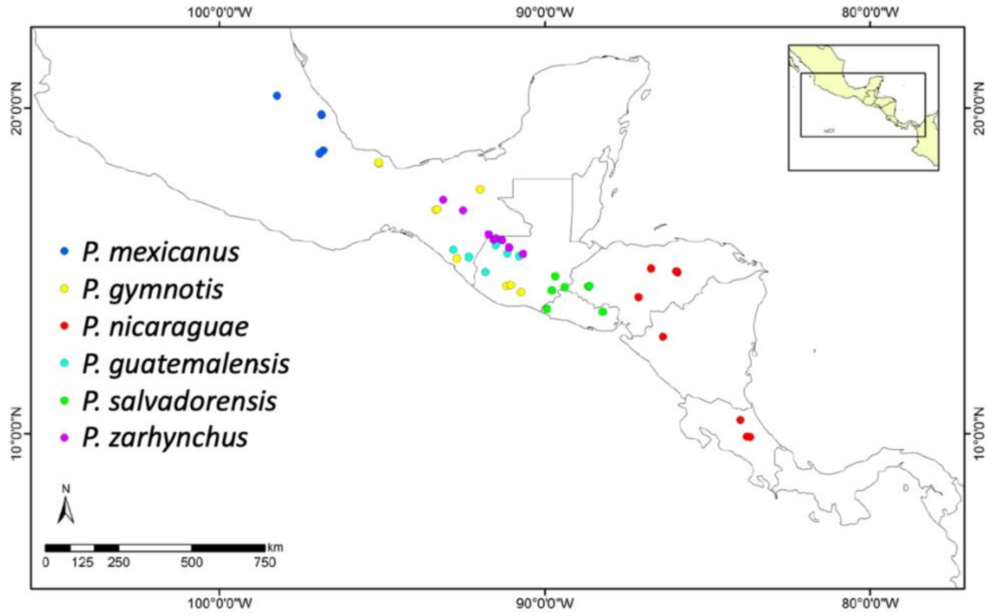

b)

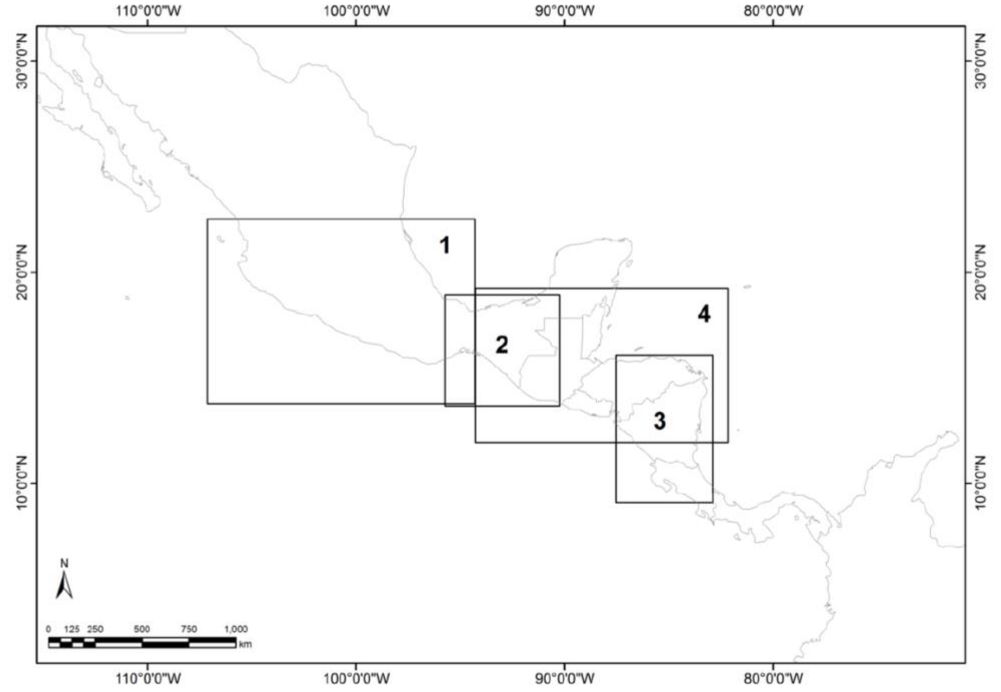

Figure S1. (**a**) Map showing the samples occurrences of six lineages of the *Peromyscus mexicanus* group analyzed, distributed in México, Guatemala, El Salvador, Honduras, Nicaragua and Costa Rica. (**b**) Four envelopes used to adequately select the area for modeling the lineages' distribution range. Numbers indicate (see Methods) 1: Mexicanus, 2: Gymnotis, 3: Nicaraguae, 4: Others.

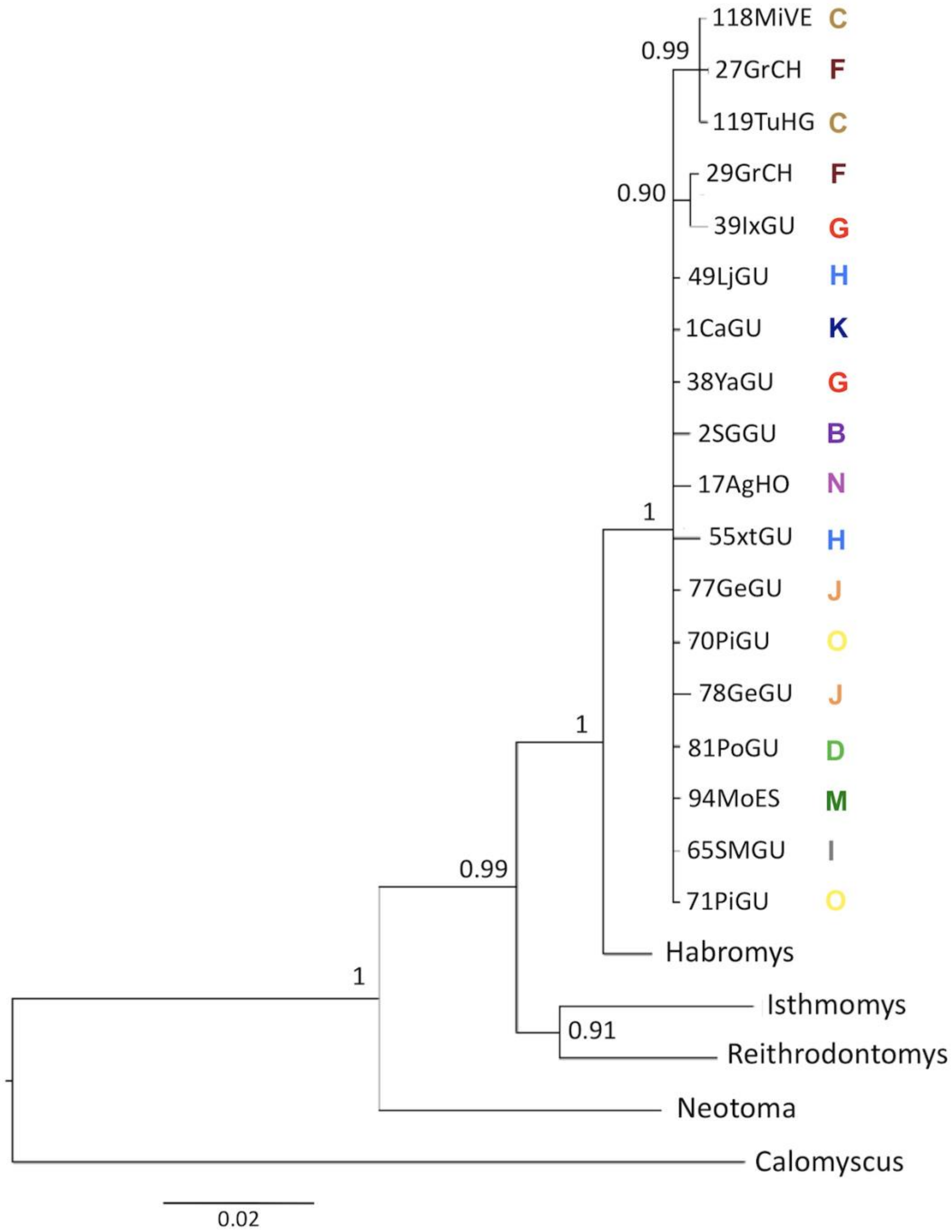

Figure S2. Bayesian inference tree based on 18 sequences (829 bp) of the Growth Hormone Receptor (GHR) nuclear gene for the *Peromyscus mexicanus* species group. These sequences represent 12 out of 15 mitochondrial lineages described in Pérez-Consuegra & Vázquez-Domínguez (2017), indicated with codes B-O. Probability values (>0.9) are indicated above branches. External group included *Calomyscus hotsoni*, *Neotoma albigula*, *Isthmomys pirrensis*, *Reithrodontomys sumichrasti* and *Habromys lophurus*. Phylogenetic tree was rooted with *Calomyscus*.

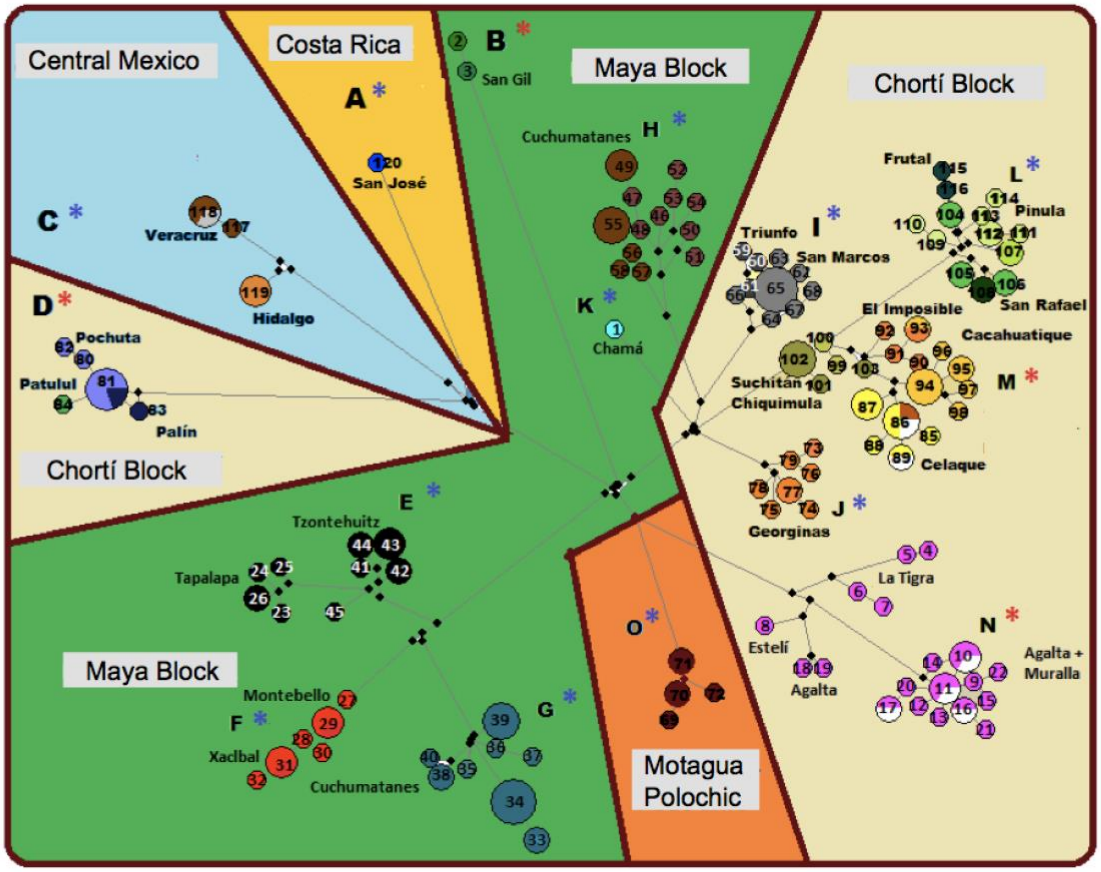

Figure S3. Haplotype network of the *Peromyscus mexicanus* species group. Size of circles is proportional to each haplotype frequency. Lineages A – O and haplotype number (following Pérez-Consuegra & Vázquez-Domínguez, 2017), name of sampling localities and physiographic regions (within gray squares) are indicated. Red and blue asterisks depict the low and midland (0-2500 m) and the highland (reaching above 3000 m) lineages, respectively.

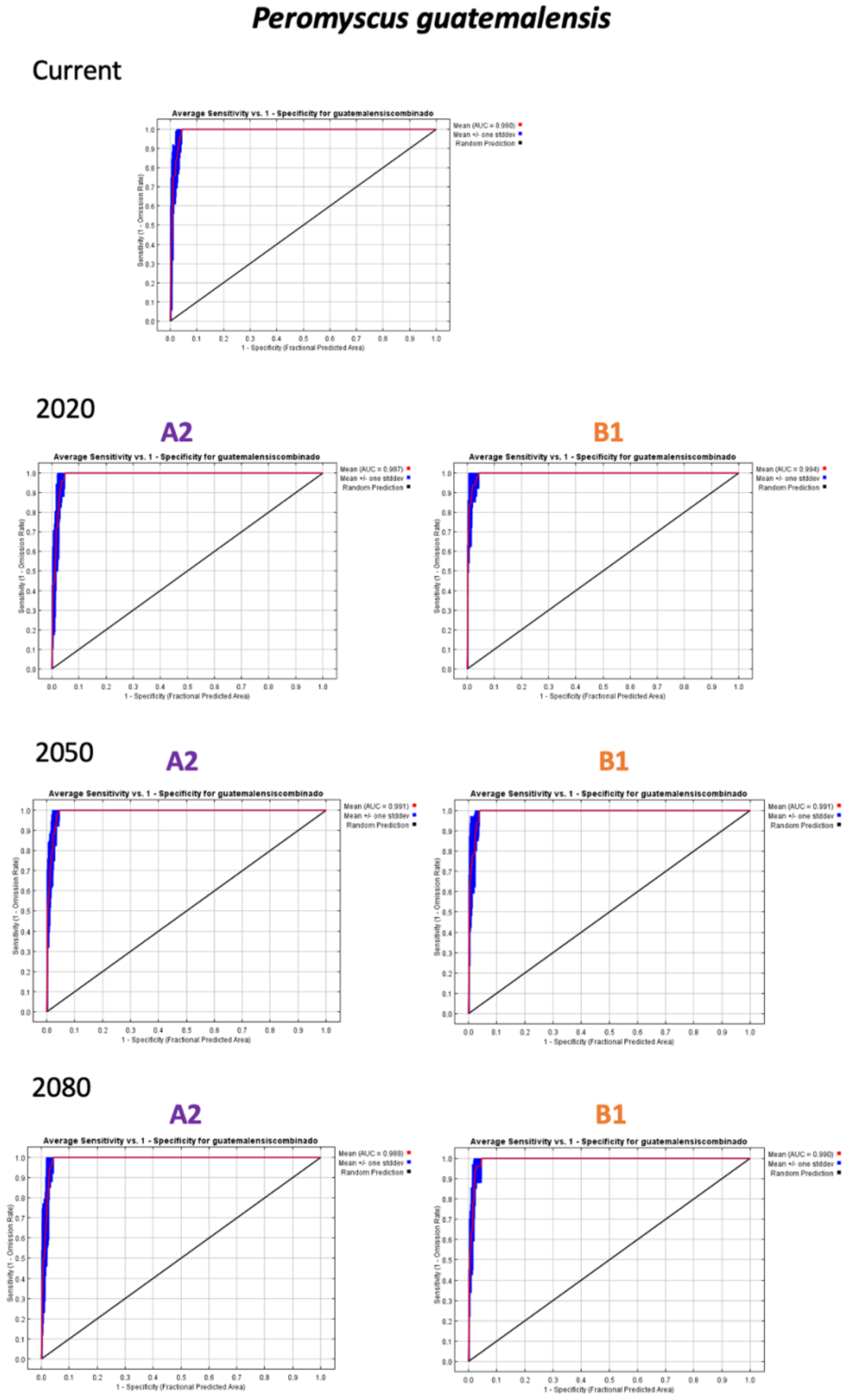

Figure S4. The average area under the curve (AUC) for *Peromyscus guatemalensis*; current and future (scenarios SREASA2 and SRESB1, and general circulation model cccma_cgcm3_1_t47 for 2020, 2050 and 2080) results are shown. The red line is the mean value for the 10 Maxent runs and the blue bar represents ± 1 standard deviation.

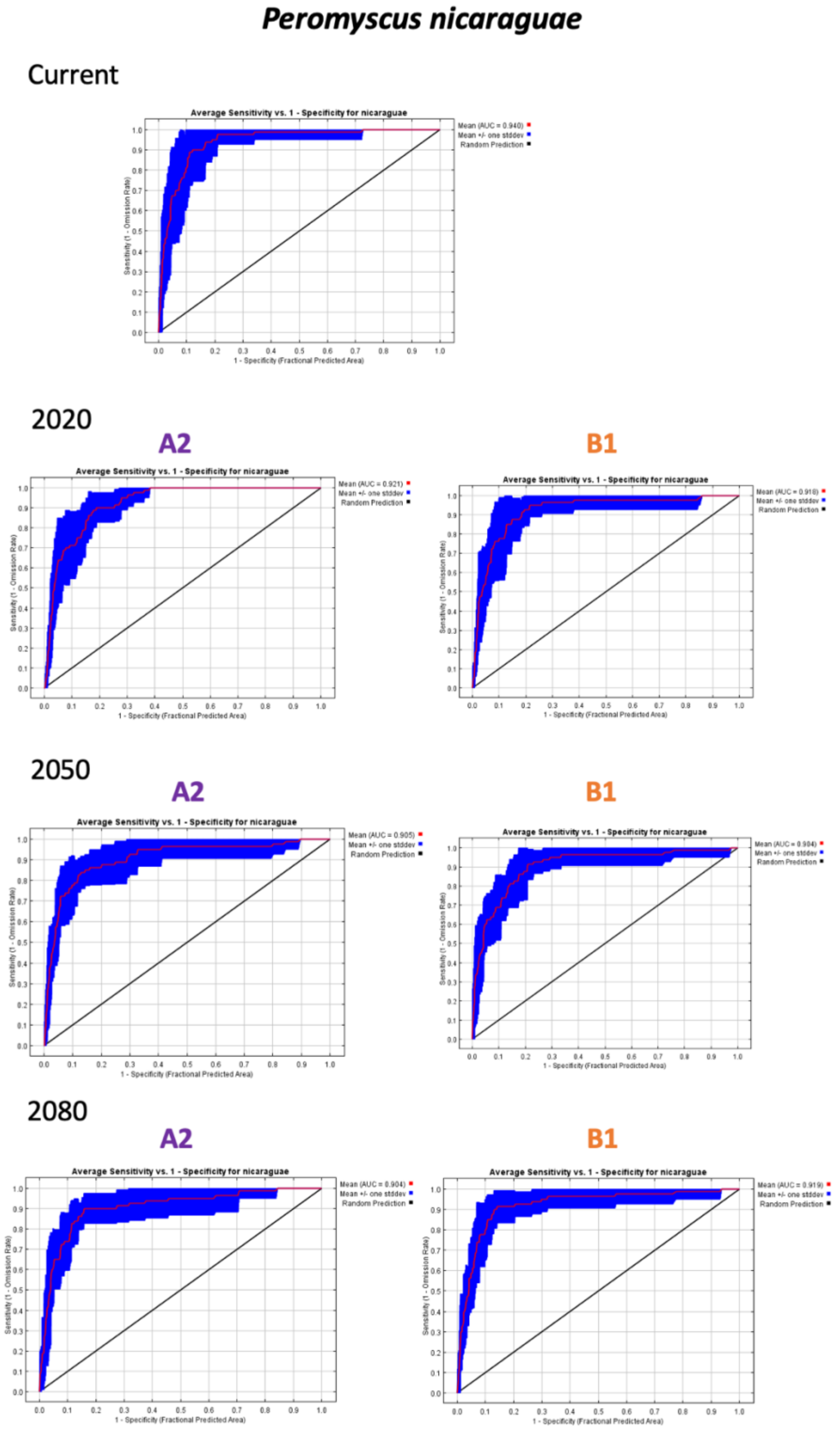

Figure S5. The average area under the curve (AUC) for *Peromyscus nicaraguae*; current and future (scenarios SREASA2 and SRESB1, and general circulation model cccma_cgcm3_1_t47 for 2020, 2050 and 2080) results are shown. The red line is the mean value for the 10 Maxent runs and the blue bar represents ± 1 standard deviation.

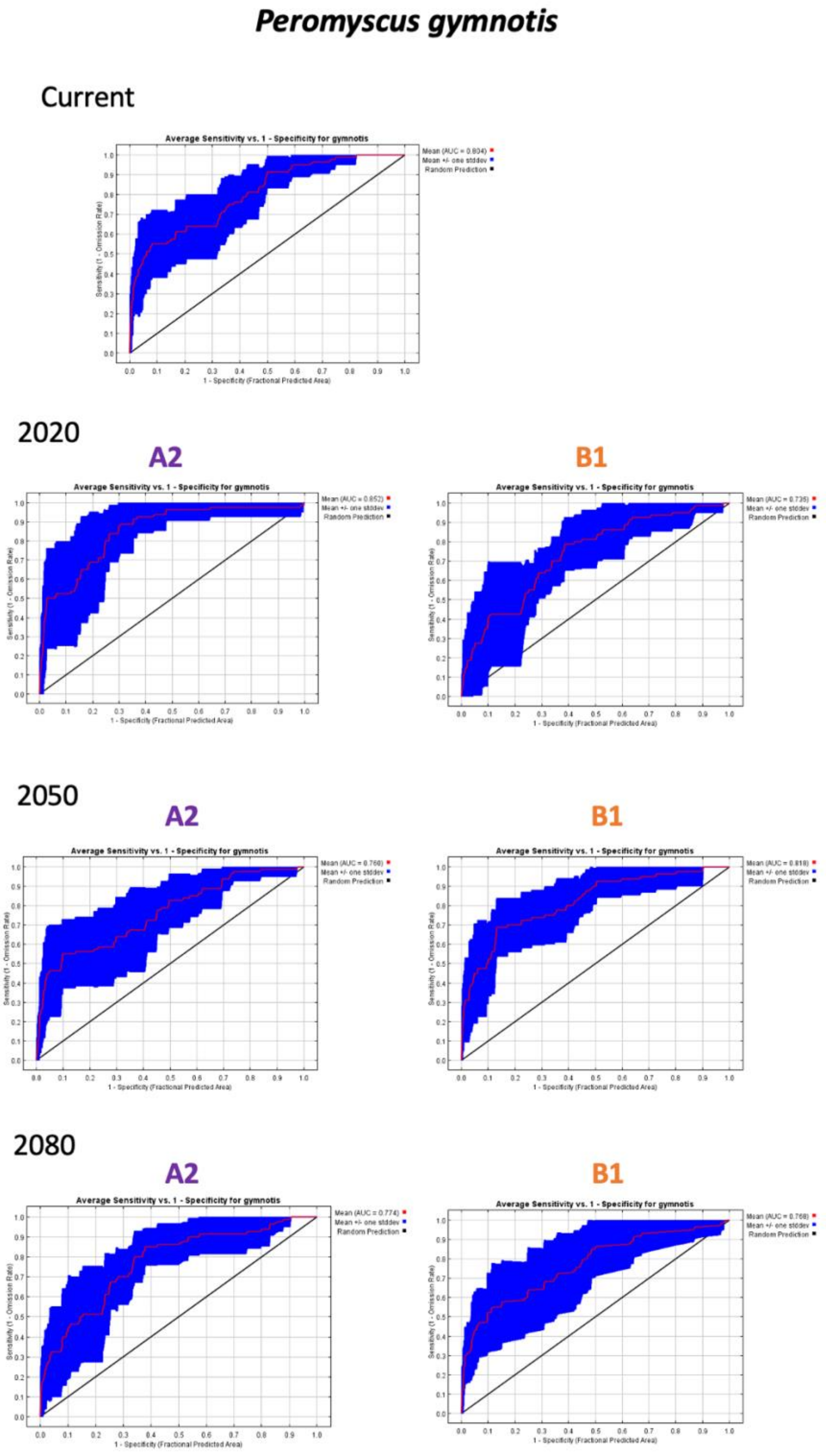

Figure S6. The average area under the curve (AUC) for *Peromyscus gymnotis*; current and future (scenarios SREASA2 and SRESB1, and general circulation model cccma_cgcm3_1_t47 for 2020, 2050 and 2080) results are shown. The red line is the mean value for the 10 Maxent runs and the blue bar represents ± 1 standard deviation.

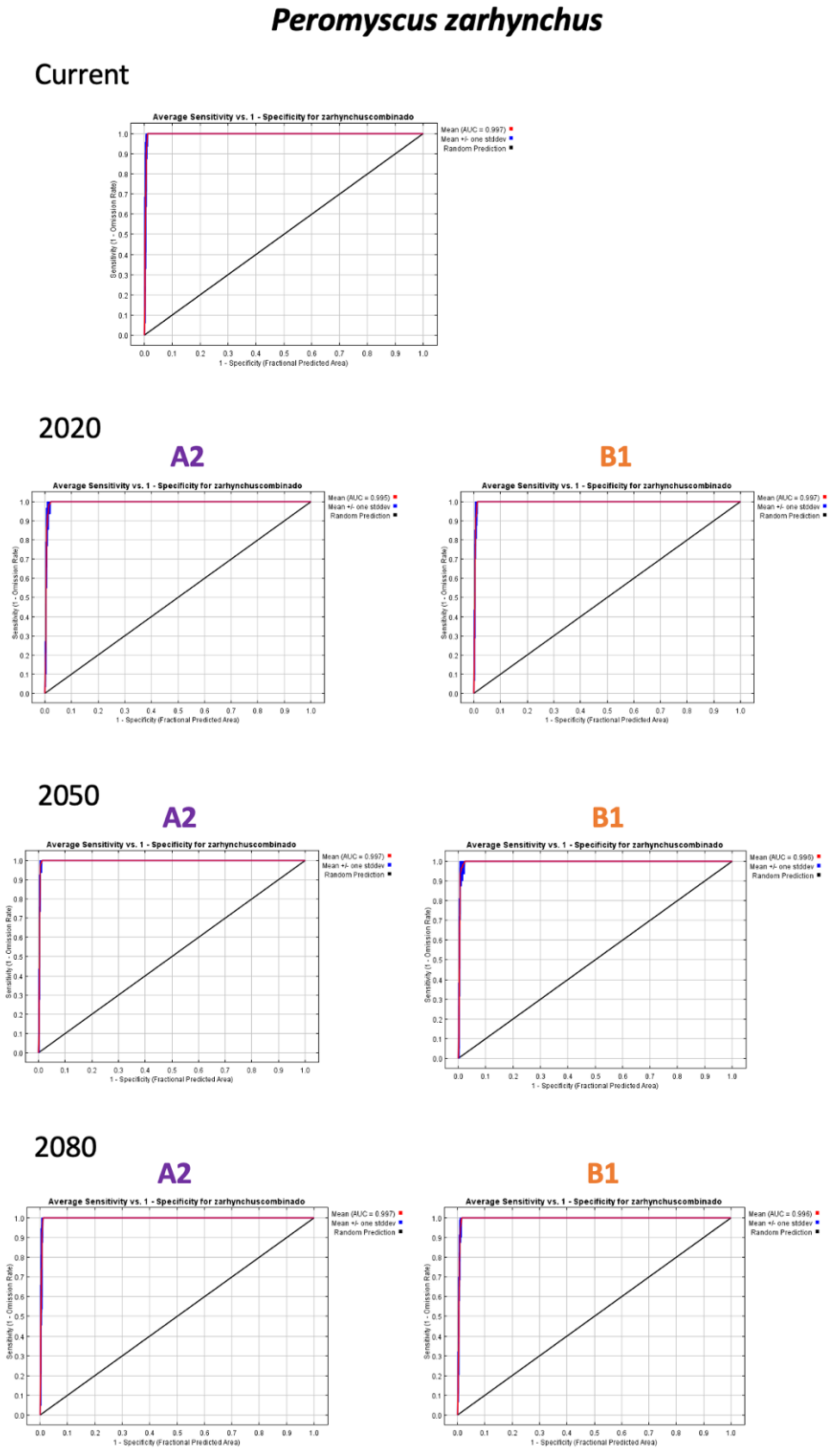

Figure S7. The average area under the curve (AUC) for *Peromyscus zarhynchus*; current and future (scenarios SREASA2 and SRESB1, and general circulation model cccma_cgcm3_1_t47 for 2020, 2050 and 2080) results are shown. The red line is the mean value for the 10 Maxent runs and the blue bar represents ± 1 standard deviation.

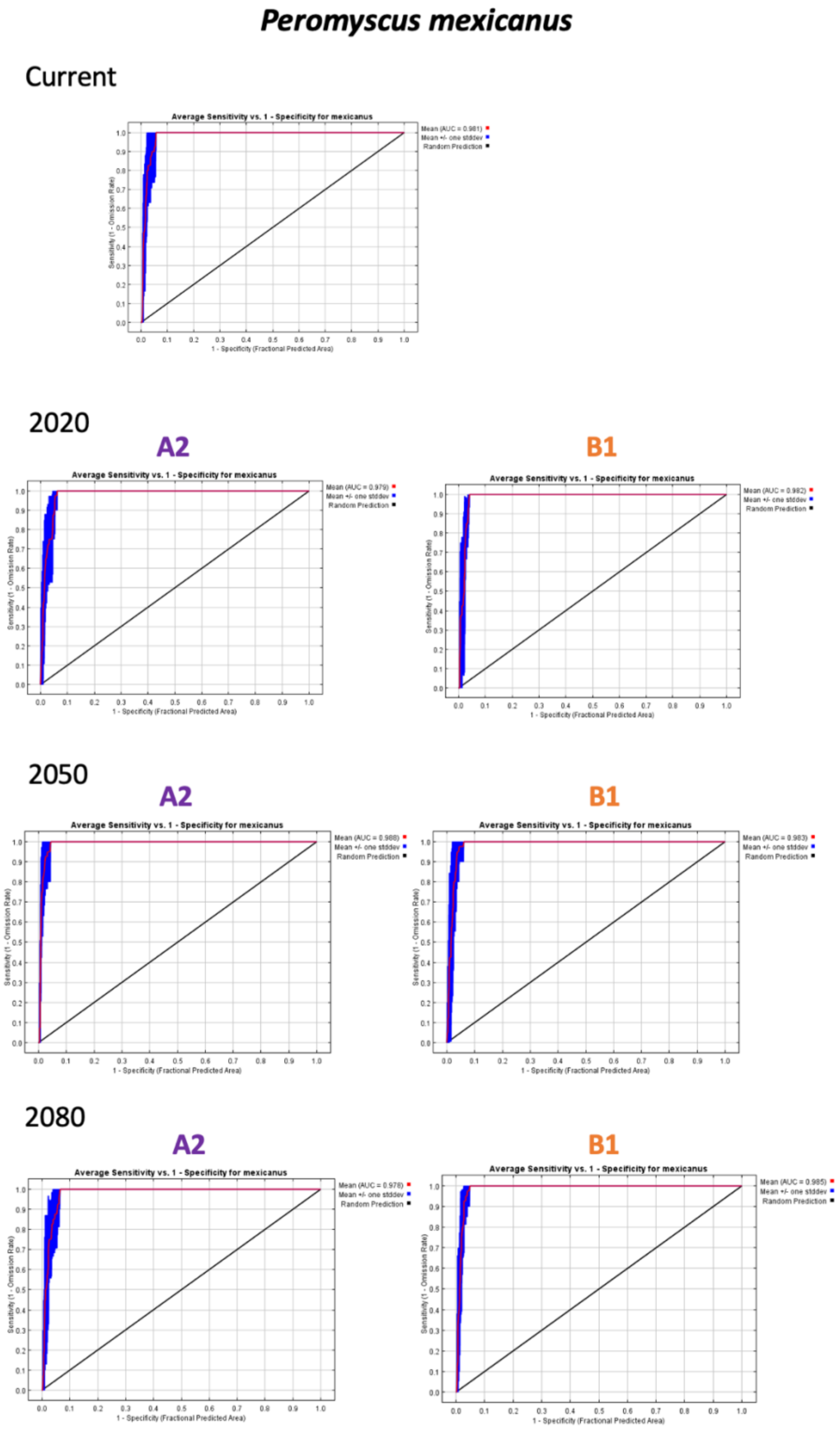

Figure S8. The average area under the curve (AUC) for *Peromyscus mexicanus*; current and future (scenarios SREASA2 and SRESB1, and general circulation model cccma_cgcm3_1_t47 for 2020, 2050 and 2080) results are shown. The red line is the mean value for the 10 Maxent runs and the blue bar represents ± 1 standard deviation.

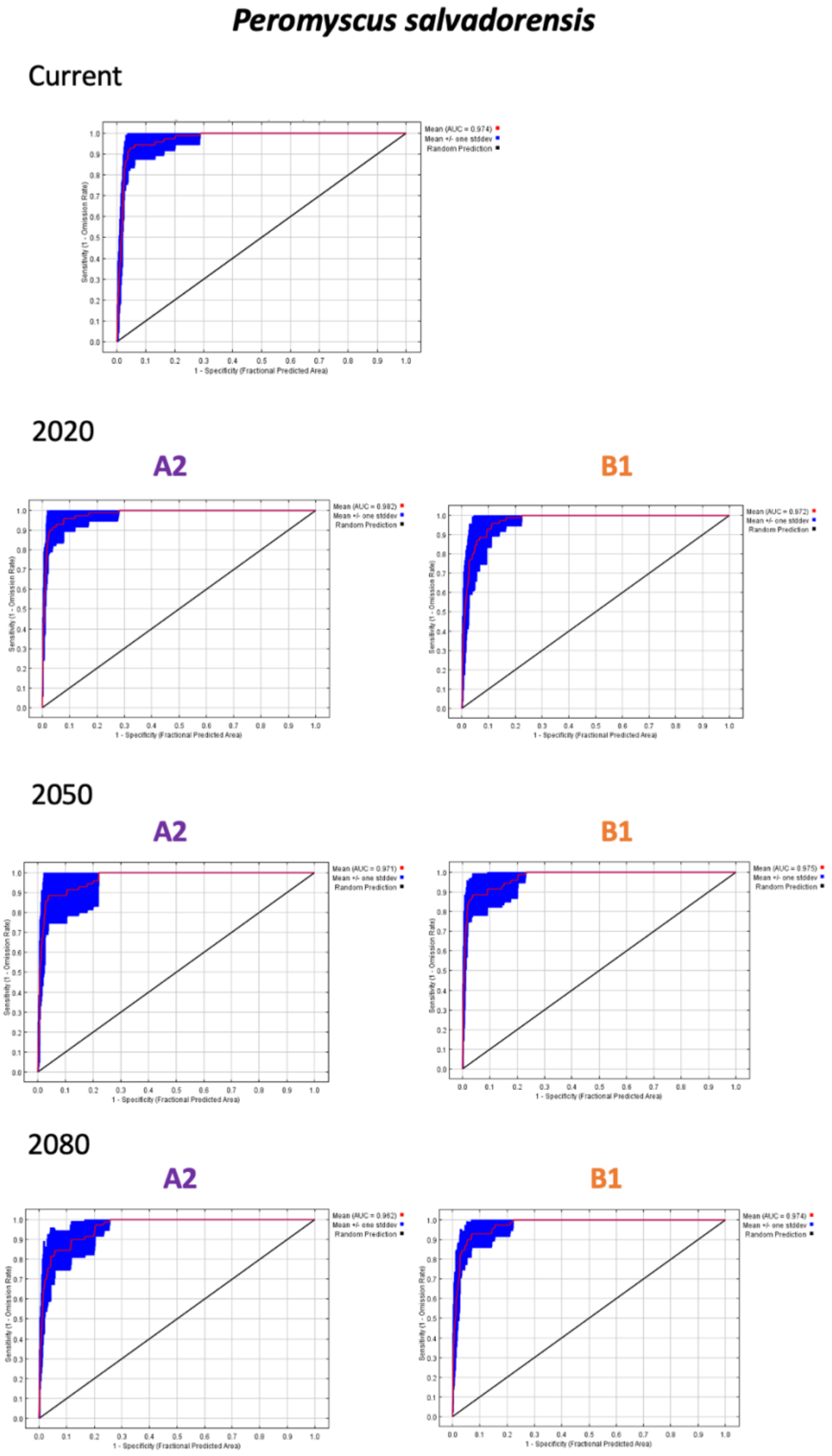

Figure S9. The average area under the curve (AUC) for *Peromyscus salvadorensis*; current and future (scenarios SREASA2 and SRESB1, and general circulation model cccma_cgcm3_1_t47 for 2020, 2050 and 2080) results are shown. The red line is the mean value for the 10 Maxent runs and the blue bar represents ± 1 standard deviation.

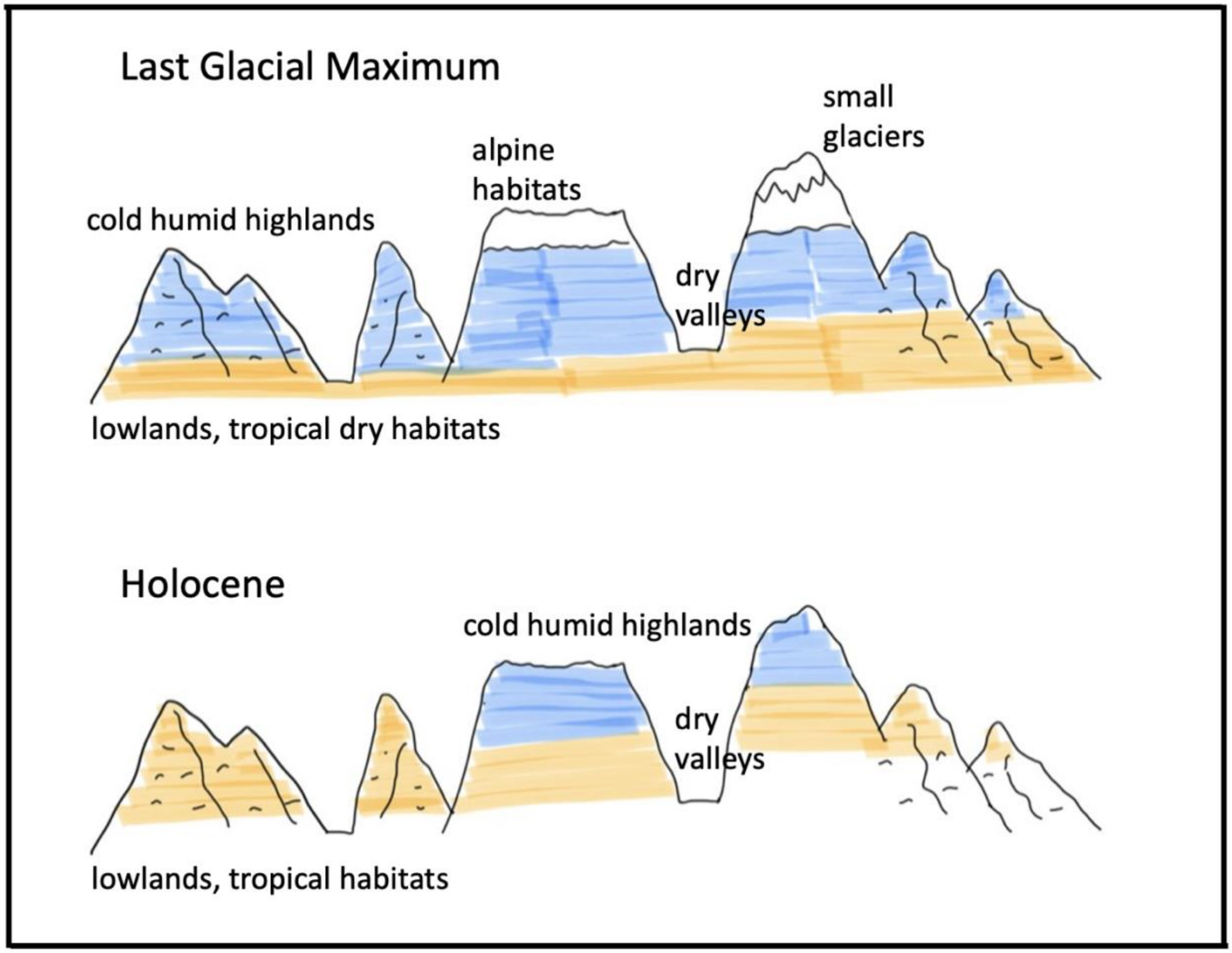

Figure S10. Hypothetic scenarios of changes in geographic ranges of the mountain mice since the Last Glacial Maximum (LGM). During LGM glacial cycles, cold humid areas occupied extensive areas jointly with reduced tropical dry lowlands. During the Holocene and towards the present, these areas changed to reduced cold habitats on mountain peaks and wide tropical habitats extending from mid to low elevations.
